# Dynamic cell responses in *Thermoanaerobacterium* sp. under hyperosmotic stress

**DOI:** 10.1038/s41598-017-10514-8

**Published:** 2017-08-30

**Authors:** Muzi Zhu, Wudi Fan, Yaping Cha, Xiaofeng Yang, Zhicheng Lai, Shuang Li, Xiaoning Wang

**Affiliations:** 1 Guangdong Provincial Key Laboratory of Fermentation and Enzyme Engineering, School of Biology and Biological Engineering, South China University of Technology, Guangzhou, China; 2 State Key Laboratory of Kidney, the Institute of Life Sciences, Chinese PLA General Hospital, Beijing, China

## Abstract

As a nongenetic engineering technique, adaptive evolution is an effective and easy-to-operate approach to strain improvement. In this work, a commercial *Thermoanaerobacterium aotearoense* SCUT27*/Δldh*-G58 was successfully isolated via sequential batch fermentation with step-increased carbon concentrations. Mutants were isolated under selective high osmotic pressures for 58 passages. The evolved isolate rapidly catabolized sugars at high concentrations and subsequently produced ethanol with good yield. A 1.6-fold improvement of ethanol production was achieved in a medium containing 120 g/L of carbon substrate using the evolved strain, compared to the start strain. The analysis of transcriptome and intracellular solute pools suggested that the adaptive evolution altered the synthesis of some compatible solutes and activated the DNA repair system in the two *Thermoanaerobacterium* sp. evolved strains. Overall, the results indicated the potential of adaptive evolution as a simple and effective tool for the modification and optimization of industrial microorganisms.

## Introduction

Microbial fermentation is continuously developed to shorten fermentation time, lower costs, and increase product yields^1^. High-sugar-medium fermentation has several advantages, including improved device efficiency, enhanced product output per unit time, decreased heat and water consumption, and simplified downstream extraction processes^2, 3^. In bioethanol production, high-concentration mash fermentation techniques are primarily used because it can increase cell density, product concentration, and production rate^2, 4^. At 25% (*w/v*) sugar medium, more than 15% (*v/v*) of ethanol can be obtained by *Saccharomyces cerevisiae*, which exhibits good tolerance to sugars and metabolites^3^.

High substrate concentration leads to elevated osmotic pressure and can thus cause cell dehydration^5^, plasmolysis^6^, and even cell inactivation^7^. These effects results in extended lag phase, metabolic disturbance, low productivity, and stagnation of fermentation^8, 9^. Thus, bacteria resistant to hypertonic solution are necessary to economic industrial production. Unfortunately, most native microorganisms cannot tolerate extreme hyperosmotic environments. Thus, processes, such as domestication, which is a relatively simple and effective method to produce tolerant mutants, is frequently employed. Through adaptive evolution and process optimization, *S*. *cerevisiae* and *Zymomonas mobilis* can utilize more than 250 g/L of glucose for ethanol production^2, 3, 5, 9^. Furthermore, an engineered *Escherichia coli* strain was adaptively evolved for xylose resistance at 120 g/L xylose and production of d-lactate is 50% higher than that by a start strain under same conditions^10^.

In the present study, the tolerance and ethanol productivity of a genetically engineered *Thermoanaerobacterium aotearoense* SCUT27/*Δldh* ^11^ subjected to a 120 g/L sugar substrate was enhanced through adaptive evolution. This bacterium can produce ethanol with high yield from lignocellulosic biomass^11, 12^. Bacterial regulatory response against high substrate conditions was further investigated.

## Results and Discussion

### Adaptive evolution under 120 g/L sugar stress

A high initial sugar concentration during fermentation is favorable because it increases fermentation efficiency at low process water and energy conditions^13^. In the present study, two high-carbon-source concentrations (81 and 120 g/L of glucose and xylose at gram ratio of 2:1) were evaluated for the modified strain SCUT27/*Δldh* ^11, 12^ in 125 mL serum bottles. After 50-h cultivation, no apparent cell growth was observed at the initial substrate concentration of 120 g/L.

**Figure 1:**
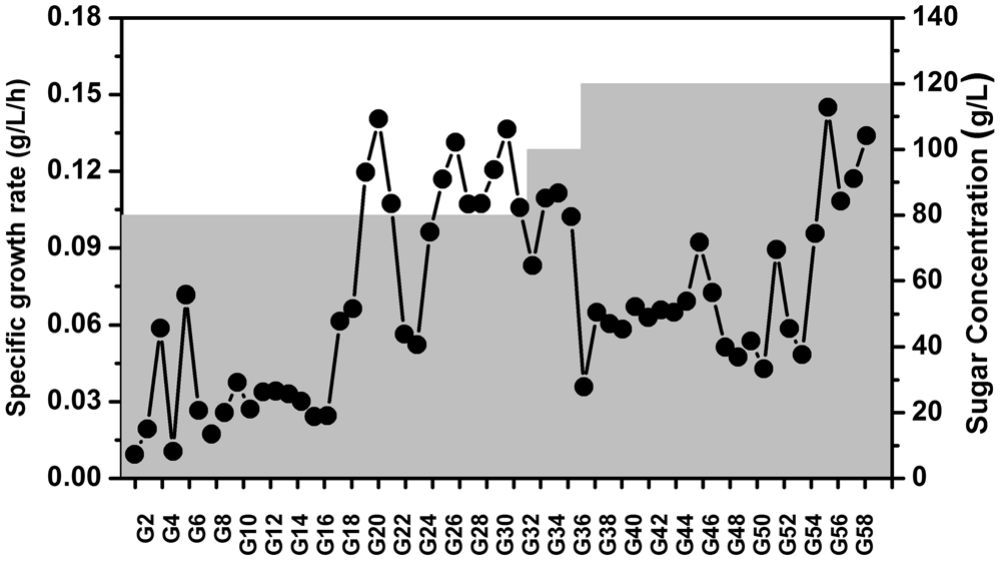
Adaptive evolution of *T*. *aotearoense* SCUT27*/Δldh* to step-increasing sugar concentration medium. Sugar was mixed by glucose and xylose at a ratio of 2:1 (g:g) and its concentration was indicated by the grey background.

The strain was subjected to a gradually increasing sugar concentration to increase the survival or reproduction of ethanol-producing strain SCUT27/*Δldh* in a medium with high initial sugar concentration. Only the individuals capable of growing under controlled environments were selected and transferred (Fig. 1). The start sugar concentration was set at 81 g/L in the evolution experiments. In the initial 16 passages, the cells had a low growth rate (approximately 0.03 g/L · h). The growth rate then showed a shock-type increase and was stabilized at 0.10–0.14 g/L · h. Meanwhile, the survival cells adapted well to 100 g/L sugar medium. The cells were then inoculated into a medium containing 120 g/L of carbon source. Low growth rate and shock-type growth increase were observed again until the cells remained larger than 0.10 g/L · h for four passages. Finally, 0.2 mL of the 58-passage culture was plated on the DSMZ 640 agar plates and incubated at 50 °C for 3 days. A total of 10 single colonies were selected and transferred into serum bottles containing 120 g/L of sugar each. After five-time transfer, only one colony exhibited steady growth in the 120 g/L medium. This colony was designated as SCUT27/*Δldh*-G58 and subjected to further research.

### Fermentation characteristics of SCUT27/*Δldh* and G58

**Figure 2:**
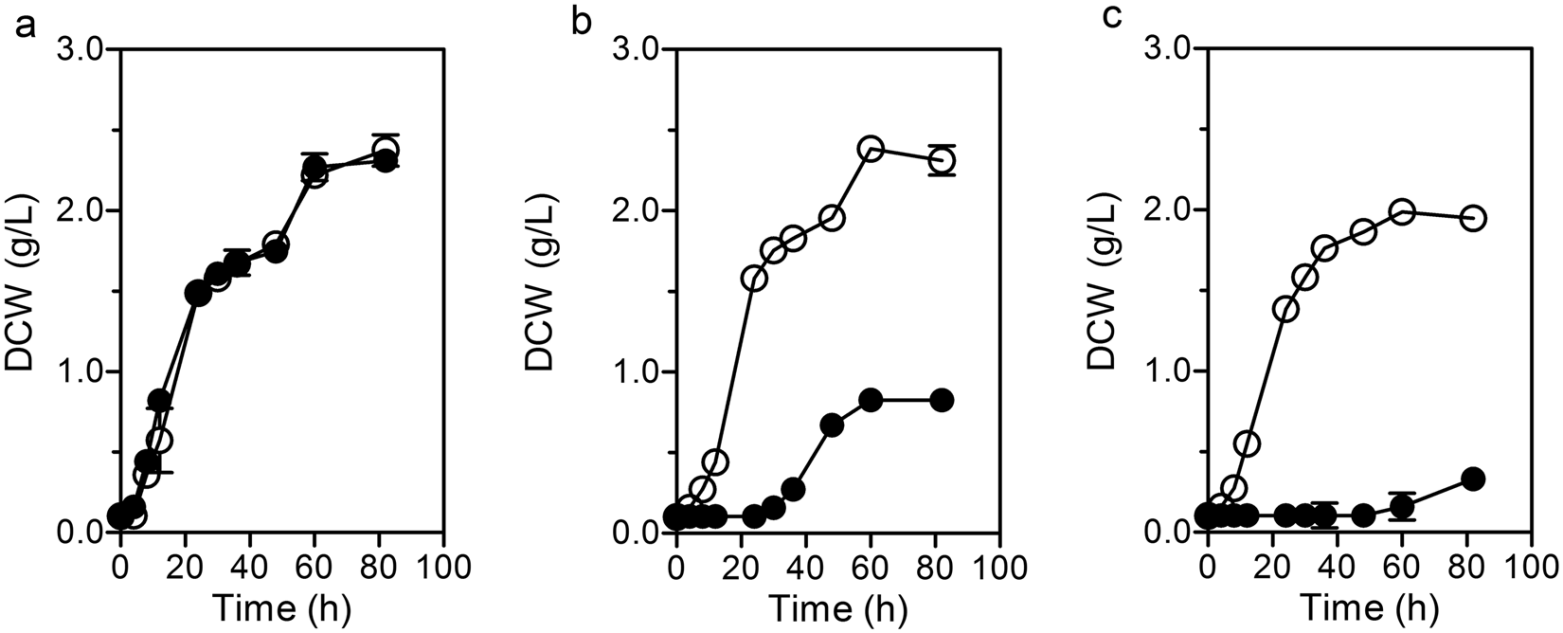
Growth profiles of *T*. *aotearoense* SCUT27/*Δldh* and G58 in three different concentration mediums. (**a**) 30 g/L, (**b**) 81 g/L and (**c**) 120 g/L. Solid circles indicated the parent strain of SCUT27/*Δldh*; hollow circles represented the evolved SCUT27/*Δldh*-G58.

For the comparison between SCUT27/*Δldh* and G58 with regard to fermentation, the strains were cultured separately with 30, 81, and 120 g/L total sugar substrate in 125 mL serum bottles, and their growth profiles were monitored (Fig. 2). In the low-sugar medium (30 g/L), the growth profiles were roughly similar to those of the start strain SCUT27/*Δldh* and its derivative G58. The strain took approximately 8 h to reach the dry cell weight (DCW) of 0.5 g/L. After 82 h incubation, the final DCW of the SCUT27/*Δldh* was only 0.8 g/L when the substrate concentration was increased to 81 g/L. However, no apparent cell growth in SCUT27/*Δldh* was observed during the 60 h cultivation at 120 g/L initial sugar concentration. The metabolite compositions of *T*. *aotearoense* SCUT27/*Δldh* and G58 were detected after a 48 h incubation (see Supplementary Table S1). For the parent strain SCUT27/*Δldh*, the concentration and yield of ethanol decreased at increased initial sugar concentration, indicating that high-sugar level can markedly inhibit cell growth and extend lag time. However, the evolved strain G58 showed substantial tolerance to high substrate concentrations. No considerable inhibition of cell growth was observed even when the initial substrate concentration was increased to 120 g/L. The cell growth curves at different concentration media clearly demonstrated that the evolved strain gained resistance to 120 g/L sugar environments. Concurrently, no change was noted in the yield of ethanol production of SCUT27/*Δldh*-G58 subjected to different concentrations of carbon source.

**Figure 3:**
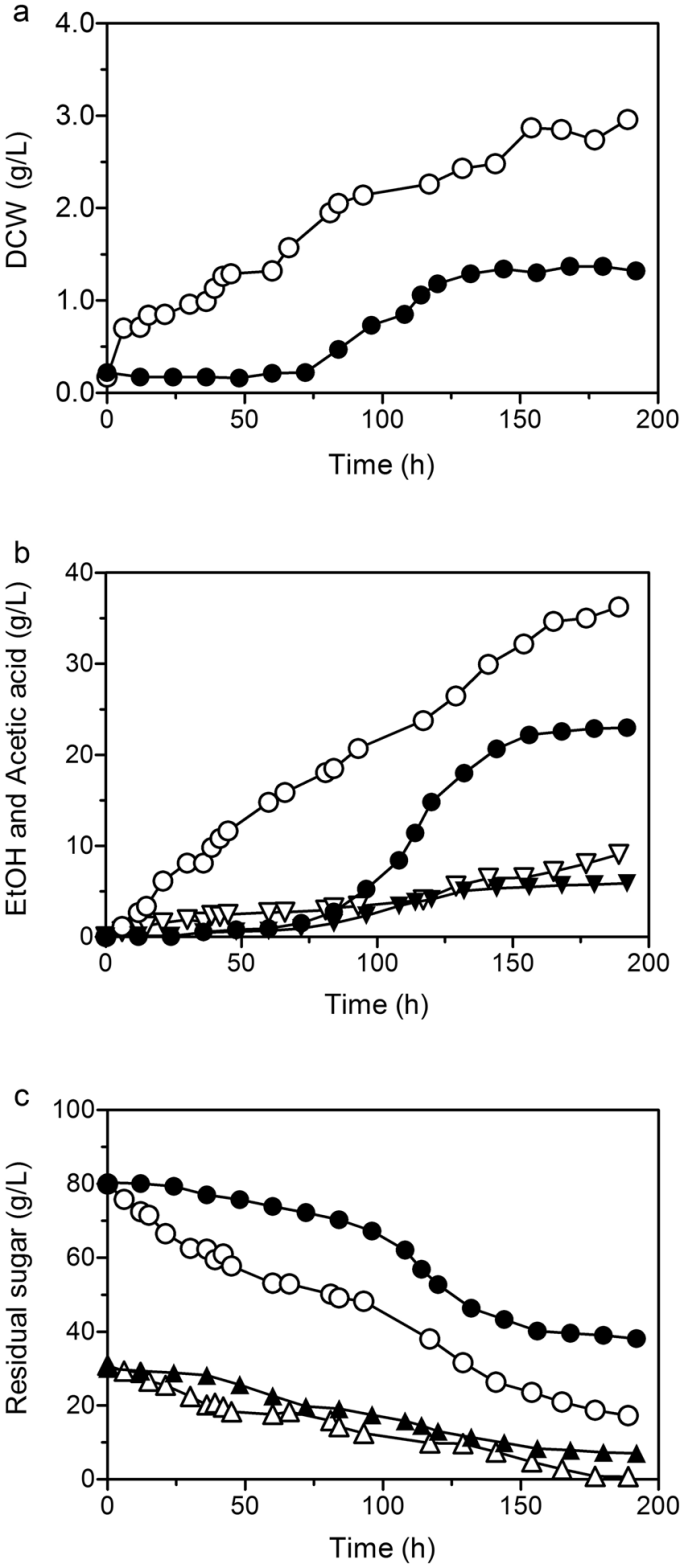
Characteristic comparison of SCUT27/*Δldh* and G58 in 5-L fermenter containing 120 g/L sugars. (**a**) Cell growth curves. (**b**) Produced ethanol (circle) and acetic acid (triangle). (**c**) Residual sugar concentration including glucose (circle) and xylose (triangle). Solid symbols represented the SCUT27/*Δldh*, and the corresponding hollow ones indicated the SCUT27/*Δldh*-G58.

The strains were cultured in a 5 L fermenter containing 2 L of medium with 80 g/L of glucose and 40 g/L of xylose to investigate the fermentation characteristics of SCUT27/*Δldh* and G58. After inoculation, cell growth was observed immediately in the strain of SCUT27/*Δldh*-G58 (Fig. 3a). By contrast, approximately 75 h lag time was required to enable the SCUT27/*Δldh* to acclimate in environments subjected to high osmotic pressures. After 200 h fermentation, the final DCW values of SCUT27/*Δldh* and G58 were approximately 1.32 and 2.96 g/L, respectively. In addition, ethanol produced by strain G58 reached 36.2 g/L, which was 1.6-fold higher than that produced by SCUT27/*Δldh* (Fig. 3b). The glucose and xylose content in G58 were consumed instantaneously, and the consumption percentages were 78.5% and 97.5%, respectively (Fig. 3c). The sugar consumption results indicated that no significant carbon catabolite repression occurred in the mixed sugar medium and thus are consistent with our previous reports^14^. Meanwhile, for SCUT27/*Δldh*, only 52.4% of initial glucose and 76.5% of initial xylose were consumed. Low sugar utilization for SCUT27/*Δldh* was in accordance with its cell growth and metabolite production. Ethanol yields were 0.35 and 0.39 g/g for the start and resultant G58 strain, respectively. It should be noted that during the last 50 hours of fermentation, almost no changes of sugar consumption, cell growth or ethanol production were recorded for SCUT27/*Δldh*. However, the corresponding sugar depletion and ethanol accumulation rates of SCUT27/*Δldh*-G58 were remained as 0.19 ± 0.05 g/L/h and 0.17 ± 0.06 g/L/h, respectively. We believe that more ethanol could be produced for SCUT27/*Δldh*-G58 if the fermentation time was extended, without considering the ethanol inhibition on the bacterium. This study demonstrated that adaptive evolution can dramatically shorten the lag time and increase the final ethanol titer in 120 g/L sugar concentration media while eliciting minimal alteration in ethanol yield (Table 1).

**Table 1:** Characteristic comparison of *T*. *aotearoense* with other reported strains.

Strain	*S. cerevisiae* NP 01	*K. marxianus* NIRE-K1	*Z. mobilis*	*E. coli* KO11 PPAL	*C. thermocellum* LL1210	*T. saccharolyticum* ALK2	*T. aotearoense* SCUT27/Δldh	*T. aotearoense* SCUT27/Δldh-G58
Carbon source	Sweet sorghum juice	Glucose	Glucose or xylose	Glucose	Cellulose, cellobiose	Glucose, xylose	Glucose, xylose, cellobiose, mannose	Glucose, xylose, cellobiose, mannose
Substrate concentration	Containing 280 g/L of total sugar	40 g/L	170 g/L glucose & 60 g/L xylose	40 g/L	60 g/L of cellulose	70 g/L xylose	20 g/L of glucose and 10 g/L of xylose	80 g/L of glucose and 40 g/L of xylose
Culture temperature/pH (°C/pH)	30/Natural pH	45/5.5	32/6	35/7	55/7	55/5.5	55/6.5	55/6.5
Fermentation mode	Batch/0.31 vvm	Batch/anaerobic	Batch/anaerobic	Batch/anaerobic	Batch/anaerobic	Continuous cultures/anaerobic	Batch/anaerobic	Batch/anaerobic
Ethanol Product (g/L)	127.8	17.73 ± 0.03	110	13.5	22.4 ± 1.4	33.1	22.9	36.2
Ethanol Yield (g/g)	0.49 ± 0.01	0.44 ± 0.01	0.47	0.37 ± 0.009	0.39	0.46	0.35	0.39
By-products	Glycerol	Acetic acid, Glycerol	Fatty acids	Acetic acid	Hydrogen	Hydrogen	Acetic acid, Hydrogen	Acetic acid, Hydrogen
Sterilization	Autoclaved at 110 °C for 40 min	Needed	Needed	Antibiotics and needed	ND^a^	ND^a^	Not needed	Not needed
Reference	9	64	5	65	20	66	This study	This study

^a^ND: not defined.

Strong tolerance to high-sugar medium enables cells to consume and use sugar rapidly, thereby rendering the strains competitive for industrial fermentation. Although genetic engineering is complex and varies in difficulty in different species, it is a direct approach that improves strain resistance to high osmotic pressures^15–17^. However, genetic manipulation in *T*. *aotearoense* SCUT27 is immature and unstable. Thus, transformation and competent cell preparation methods, such as electroporation^11^ and natural competence cell^18^, were employed by our group to transfer exogenous DNA into *T*. *aotearoense* SCUT27. However, only a few of these methods worked^11, 19^, and successful results were not reproduced. Another simple method of obtaining tolerant strains is performing long-term adaptation studies. Liang *et al*.^20^ employed adaptive evolution to increase the tolerance of *C*. *thermocellum* to cellobiose, reaching the highest concentration of 50 g/L after 13 weeks. They were able to produce 22.4 g/L of final ethanol by using 60 g/L of cellulose as substrate. *E*. *coli* can be cultured in a medium with gradually increasing ethanol content. Some studies reported that evolved strains have enhanced resistance to ethanol and decreased sensitivity to toxic aldehydes after 3 months of cultivation^21, 22^. In a previous study, *T*. *aotearoense* LA1002 was domesticated to adapt to high sugar concentration^23^. The results showed that the evolved strain can survive and ferment in 100 g/L of substrate (90 g/L glucose and 10 g/L fructose) for lactic acid production. The LA1002-G40 was used to produce lactic acid from mixed bakery waste hydrolysates, yielding 0.85 g/g. In the present study, resistance to sugar concentration was enhanced to 120 g/L over a 1.5-month period for *T*. *aotearoense* SCUT27/*Δldh* fermentation. Moreover, the resultant strain improved xylose utilization and ethanol productivity. Thus, this tolerance phenotype of SCUT27/*Δldh*-G58 has potential in lignocellulosic biomass fermentation.

### Propagation stability of evolved strain

The phenotype stability of SCUT27*/Δldh*-G58 was examined by passing on from transfer to transfer in 120 g/L mixed sugar medium. After the strain was subcultured for 40 times, standard deviation values of different culture transfer in cell growth and metabolite spectrum were lower than 5%, indicating no significant change. This result showed that SCUT27*/Δldh*-G58 is resistant to high sugar conditions and thus can be used for ethanol production.

### Transcriptome and intracellular solute pools analysis

Adaptive evolution enables SCUT27/*Δldh*-G58 to have a short lag phase, fast sugar consumption, and high ethanol production in a 120 g/L sugar medium. However, the regulatory mechanisms involved in the tolerance of SCUT27/*Δldh*-G58 to high-substrate medium remain unidentified. Thus, the total RNAs of SCUT27/*Δldh* and G58 were extracted for sequencing and transcriptome analysis. Concurrently, another adaptation-evolved strain of *T*. *aotearoense* LA1002-G40 for lactic acid production^23^ was analyzed. The RNAseq data have been deposited in the GenBank database under the accession number PRJNA385555 (SCUT27/*Δldh* vs G58) and PRJNA385757 (LA1002 vs G40).

According to the analysis report of Genewiz, the total number of differential expression genes (|log(change fold)| ≥ 2.0, FDR ≤ 0.05) in LA1002-G40 was higher than that in SCUT27/*Δldh*-G58 (80 vs. 53, see Supplementary Tables S2 and S3). In SCUT27/*Δldh*-G58, 33 and 20 genes were upregulated and downregulated, respectively. In LA1002-G40, 52 genes were upregulated and 28 genes were downregulated. For the evaluation of the RNA sequencing results, change folds in the transcript abundance of selected genes were assessed via RT-qPCR. Both the RNA-seq and RT-qPCR results displayed the same direction (upregulation or downregulation) of differential genes (Table 2). Altered genes were enriched into 41 and 20 pathways in LA1002-G40 and SCUT27/*Δldh*-G58, respectively, as indicated by the function analysis results.

**Table 2:** qRT-PCR results of SCUT27/*Δldh*-G58 and LA1002-G40.

Pathway	Gene_ID	Functional annotation	FDR^a^	RNA-Seq^b^	qRT-PCR^c^
**SCUT27/** ***Δldh*** **-G58 vs SCUT27/** ***Δldh***					
ko00052: Galactose metabolism	*Tsac_1294*	Galactose-1-phosphate uridyltransferase	2.55E-05	15.541	6.494 ± 1.230
	*Tsac_1295*	Galactokinase	2.38E-03	7.989	2.780 ± 0.085
	*Tsac_1296*	UDP-glucose 4-epimerase	1.51E-02	5.780	3.061 ± 0.270
ko02010: ABC transporters	*Tsac_0150*	ABC-type transporter, integral membrane subunit	1.07E-02	7.972	1.450 ± 0.283
	*Tsac_0151*	Extracellular solute-binding protein family 1	2.38E-03	8.140	1.668 ± 0.139
	*Tsac_2231*	Extracellular ligand-binding receptor	6.67E-03	8.017	1.304 ± 0.023
ko00250: Alanine, aspartate and glutamate metabolism	*Tsac_2232*	Alanine dehydrogenase	9.75E-04	11.385	1.481 ± 0.051
ko00910: Nitrogen metabolism	*Tsac_2029*	Glutamine synthase catalytic region	2.80E-02	5.141	1.182 ± 0.115
ko00564: Glycerophospholipid metabolism	*Tsac_1205*	FAD dependent oxidoreductase	4.26E-02	0.189	0.786 ± 0.068
**LA1002-G40 vs LA1002**					
ko00052: Galactose metabolism	*Tsac_1476*	UTP-glucose-1-phosphate uridyltransferase	2.93E-03	6.936	7.906 ± 0.109
	*Tsac_0599*	NAD-dependent epimerase/dehydratase	7.11E-03	5.800	14.410 ± 0.232
	*Tsac_2297*	ROK family protein	9.98E-03	5.740	5.148 ± 0.293
	*Tsac_1517*	beta-phosphoglucomutase	4.74E-03	0.160	0.212 ± 0.008
ko00250: Alanine, aspartate and glutamate metabolism	*Tsac_2392*	Asparagine synthase (glutamine-hydrolyzing)	1.18E-09	47.472	122.191 ± 5.701
ko00230: Purine metabolism	*Tsac_2588*	Inosine-5′-monophosphate dehydrogenase	1.14E-02	5.177	8.658 ± 0.311
	*Tsac_0104*	5′-Nucleotidase domain-containing protein	1.84E-03	0.137	0.096 ± 0.015
ko00290: Valine, leucine and isoleucine biosynthesis	*Tsac_0569*	Dihydroxy-acid dehydratase	1.56E-02	4.833	4.173 ± 0.527
	*Tsac_0564*	Ketol-acid reductoisomerase	1.58E-02	4.817	4.216 ± 0.296
	*Tsac_0566*	3-isopropylmalate dehydratase large subunit	1.89E-02	4.649	4.266 ± 0.252
	*Tsac_2182*	Glu/Leu/Phe/Val dehydrogenase dimerization region	2.81E-02	0.009	7.542 ± 2.795
ko00620: Pyruvate metabolism	*Tsac_0628*	Formate acetyltransferase	2.11E-02	4.560	6.277 ± 0.182
ko00051: Fructose and mannose metabolism	*Tsac_0691*	Glycoside hydrolase family 26	8.68E-03	0.184	0.219 ± 0.016
	*Tsac_0227*	Class II aldolase/adducin family protein	1.50E-02	0.198	0.303 ± 0.014
	*Tsac_2504*	PTS system mannose/fructose/sorbose family IID component	3.22E-02	0.166	0.301 ± 0.032
ko00550: Peptidoglycan biosynthesis	*Tsac_2391*	Penicillin-binding protein, 1A family	1.07E-04	11.058	15.423 ± 0.995
	*Tsac_1570*	UDP-N-acetylglucosamine 1-carboxyvinyltransferase	1.67E-02	4.807	5.905 ± 0.793
ko00910: Nitrogen metabolism	*Tsac_1487*	FAD-dependent pyridine nucleotide-disulfide oxidoreductase	5.92E-04	0.117	0.114 ± 0.016

^a^False discovery rate.

^b^The change fold in gene expression obtained by RNA-sequencing results.

^c^The change fold in gene expression obtained by qRT-PCR results. The data were mean ± standard deviation (SD) of three independent biological replicates.

#### Enhancing UDP-glucose synthesis to improve intracellular osmotic pressure

**Figure 4:**
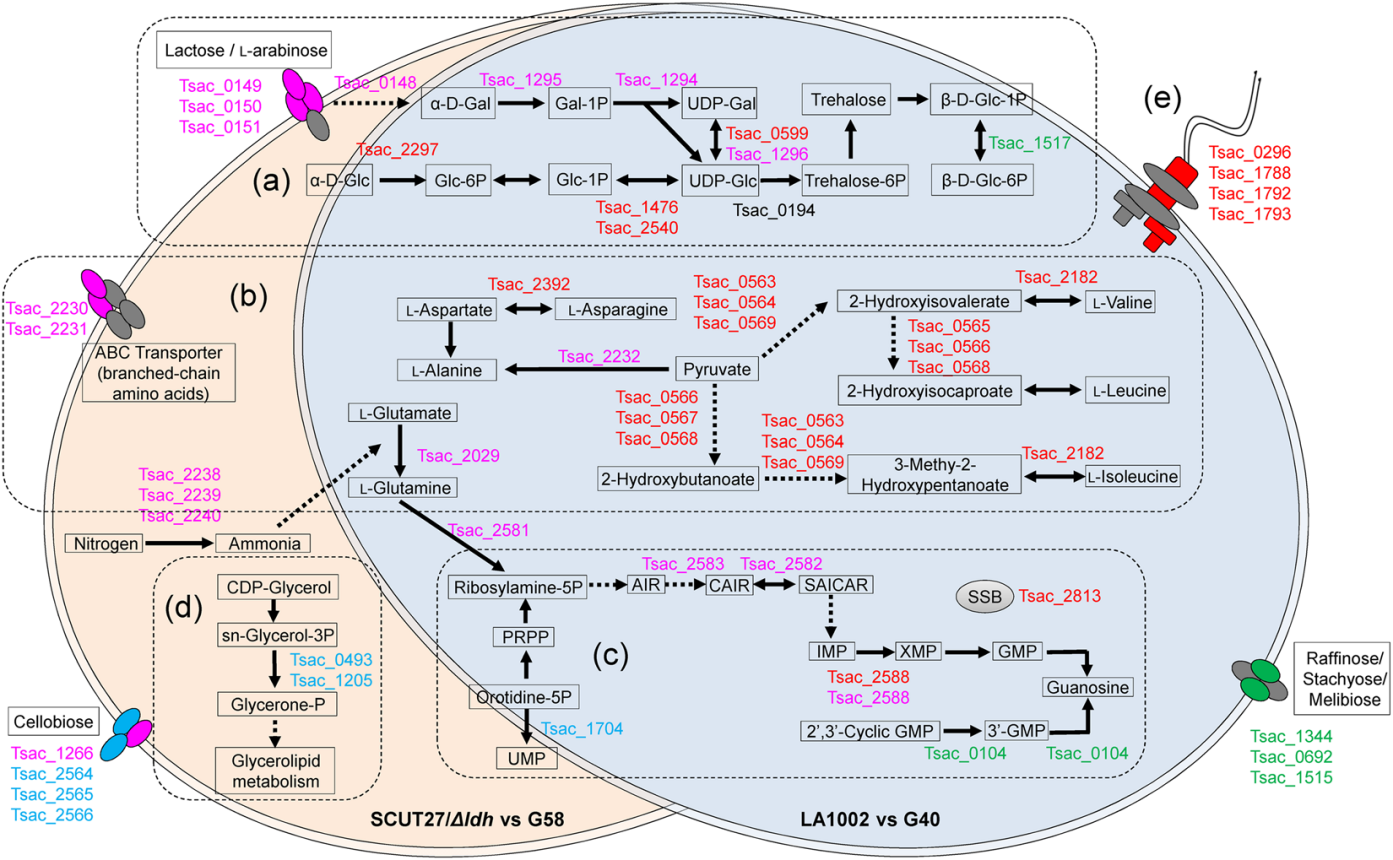
Summary of regulation profiles in *T*. *aotearoense* SCUT27/*Δldh* vs. G58 and LA1002 vs. G40. Purple symbols, up-regulated genes in SCUT27/*Δldh*-58; blue symbols, down-regulated genes in SCUT27/*Δldh*-G58; red symbols, up-regulated genes in LA1002-G40; green symbols, down-regulated genes in LA1002-G40; solid arrows, direct relations; dashed arrows, indirect relations. (**a**) Galactose metabolism pathway. (**b**) Amino acid accumulation pathway. (**c**) DNA repair regulation. (**d**) Glycerol accumulation pathway. (**e**) Flagellum assembly. UDP, Uridine diphosphate; sn-Glycerol-3P, Glycerophosphoric acid; PRPP, 5-Phospho-alpha-D-ribose 1-diphosphate; UMP, Uridine monophosphate; AIR, Aminoimidazole ribotide; CAIR, 1-(5-Phospho-D-ribosyl)-5-amino-4-imidazolecarboxylate; SAICAR, 1-(5′-Phosphoribosyl)-5-amino-4-(N-succinocarboxamide)-imidazole; IMP, Inosine 5′-phosphate; XMP, Xanthosine 5′-phosphate; GMP, Guanosine 5′-phosphate.

The transcriptional profiles of SCUT27/*Δldh* versus G58 and LA1002 versus G40 both showed that several genes involved in galactose metabolism pathway (ko00052) were upregulated after adaptive evolution (Table 2). However, the related specific genes were not consistent in the two profiles. In the SCUT27/*Δldh* versus G58 profile, the differentially expressed genes included *Tsac_1294*, *Tsac_1295*, *Tsac_0148*, and *Tsac_1296*, which can facilitate the increase in the flux of lactose to UDP-galactose (Fig. 4a). Meanwhile, in the LA1002 versus G40 profile, the differentially expressed genes were *Tsac_1476*, *Tsac_2540*, and *Tsac_2297*, which are beneficial to metabolic flux improvement from glucose to UDP-glucose. *Tsac_1296* and *Tsac_0599* are isoenzymes upregulated in SCUT27/*Δldh* versus G58 and LA1002 versus G40 profiles, respectively. Notably, the significant downregulation of *Tsac_1517* in LA1002 versus G40 profile may reduce consumption and maintain high trehalose levels in cells. As the direct precursor of trehalose synthesis^24^, the intracellular UDP-glucose concentration was determined. It was recorded as 0.0362 ± 0.0024% and 0.0199 ± 0.0017% of dry cell weight for SCUT27/*Δldh*-G58 and SCUT27/*Δldh*, respectively. Similarly, intracellular UDP-glucose was also increased by 1.88-fold from 0.0230 ± 0.0021% (LA1002) to 0.0432 ± 0.0038% (LA1002-G40) of dry cell weight. The improvement facts of intracellular UDP-glucose were consistent with the transcriptome analysis results, and partially supported the upregulation of intracellular trehalose levels in the evolved strain.

To respond to the extracellular environment changes, organisms constantly transmitted the external signals to control gene expression patterns so that cells could adjust the levels of several metabolites^25^. Among them, proper intracellular glucose partitioning plays a significant role in maintaining cellular homeostasis^26^. As the sole glucosyl donor for crucial metabolites, UDP-glucose locates at a strategic point in glucose partitioning^24, 27^. It is involved in the synthesis of many structural and storage polysaccharides, such as trehalose and β-glucan in yeast^24^, sucrose and cellulose in plant^28^, and glycogen in animals^29^. UDP-glucose pyrophosphorylase (UGPase, *Tsac_1476*, *Tsac_2540*) catalyzes the formation of UDP-glucose from glucose 1-phosphate (Glc-1P, Fig. 4) and UTP^30^. Previous studies demonstrated that the regulation of UGPase expression is related to stress response and long-term survival of yeast cells^31^. And the storage of carbohydrates, glycogen and trehalose, have been considered as the contribution of stress response^32^. In particular, trehalose plays an important role as a protectant of protein integrity against hyperosmotic, heat and oxidants stress^33–35^. Taking into consideration that UDP-glucose is the necessary supply of glucosyl for biosynthesis of other carbohydrates, the upregulation of *Tsac_1476*, *Tsac_2540* expression and concentration of UDP-glucose in the evolved strain, is based on anticipated outcome.

#### Increasing amino acid concentration in cells

When exposed to hypertonic surroundings, cells accumulate amino acids to resist high-sugar conditions through several pathways^36–38^. In SCUT27/*Δldh* versus G58 profile, *Tsac_2230*, *Tsac_2231*, encoding an ABC transporter responsible for the organic acids produced in branched-chain amino acid catabolism (Fig. 4b), were significantly upregulated by 18.752- and 8.017-fold, respectively (Table 2). Similarly, genes related branched-chain amino acid metabolism in the LA1002 versus G40 profile, were also upregulated by 4.20- to 5.20-fold (Fig. 4b). Metabolomics study has demonstrated that 2-hydroxyisovalerate, 2-hydroxyisocaproate, and 2-hydroxy-3-methylvalerate were the most robustly increased metabolites in response to osmotic stress^38^. All three compounds are 2-hydroxy carboxylic acid derivatives of the branched-chain amino acids valine, leucine, and isoleucine, respectively, and ultimately exported from the cell by the ABC transporter^39^. Intracellular amino acid concentration measurement showed that no obvious changes in the branched-chain amino acids were detected (Table 3).

**Table 3:** Effect of high osmotic stress on the intracellular amino acid in *T*. *aotearoense* ^*a*^.

Amino Acids	Intracellular content (mg/mg of cell dry weight, %)					
	*Δldh*	*Δldh*-G58	Ratio^*b*^	LA1002	LA1002-G40	Ratio^*b*^
Asp	0.030	0.252	8.50	0.113	0.112	0.99
Lys	0.061	0.092	1.49	0.071	0.108	1.52
Thr	0.056	0.082	1.45	0.069	0.066	0.96
Glu	0.291	0.795	2.74	0.238	0.690	2.90
Pro	0.014	0.021	1.58	0.016	0.036	2.26
Arg	0.034	0.022	0.65	0.030	0.048	1.57
Gly	0.040	0.071	1.80	0.059	0.084	1.42
Cys	0.099	0.147	1.48	0.090	0.090	1.00
His	0.006	0.011	1.98	0.012	0.024	1.97
Tyr	0.010	0.027	2.74	0.016	0.024	1.50
Ser	0.018	0.020	1.07	0.056	0.037	0.66
*Val* ^*c*^	0.067	0.070	1.04	0.064	0.087	1.36
*Leu* ^*c*^	0.047	0.035	0.75	0.055	0.063	1.15
*Ile* ^*c*^	0.084	0.055	0.65	0.078	0.055	0.71
Ala	0.208	0.181	0.87	0.213	0.216	1.01
Phe	0.043	0.030	0.69	0.114	0.107	0.94
Met	0.414	0.260	0.63	0.447	0.305	0.68

^*a*^The data are the means of triple experiments, and the relative standard deviation were less than 10%. Gln, Trp and Asn concentrations were not determined.

^*b*^Ratio was calculated as the amino acid content in the evolved strain (*Δldh*-G58 and LA1002-G40) divided by that in the original strain (*Δldh* and LA1002).

^*c*^Branched-chain amino acids means the sum of Val, Ile and Leu, marked as italic.

In this study, several amino acids, Asp, Glu, Pro, Gly, His and Tyr were accumulate to high concentrations in response to osmotic stress (Table 3). Meanwhile, we found that the contents of intracellular Asp and Glu were the highest in the both evolved strains. It was reported that Glu was the direct biosynthetic precursor for Pro and Arg^40^. Similarly, Asp was the precursor of Lys and Thr, and Gly was the Cys synthesis precursor, respectively^41^. As important compatible solutes, glutamate, aspartate and glycine play important roles in physiological adaptation to high external osmolarity^37, 42^. Glycine addition can increase the survival rate of *S*. *cerevisiae* in a hyperosmotic medium^43^. Pro accumulation is the primary defense response by protecting membrane from damage when plants adapt to salt stress^36, 42^. Asp and Glu could increase unsaturated fatty acids synthesis, enhance lipid transport and improve cytomembrane fluidity^44, 45^. And the permeability and fluidity of cell membrane were related to the resistance to turgor pressure in *A*. *pasteurians* ^46^. All these supported that the increase in levels of Asp, Glu and Gly and their derivative amino acids was quantitatively most important.

#### Activation of DNA repair

DNA repair regulation was complex in the two transcriptional profiles (Fig. 4c). In the SCUT27/*Δldh* versus G58 profile, three genes and one gene were upregulated and downregulated, respectively. These genes were involved in nitrogen metabolism (ko00910), glutamate metabolism (ko00250), and purine metabolism (ko00230) according to KEGG Pathway database. All the regulation may lead to increased synthesis of xanthosine monophosphate (XMP), which is the precursor of guanosine monophosphate (GMP)^47, 48^. A similar phenomenon of gene regulation for GMP accumulation was observed in the LA1002 versus G40 profile.

*Tsac_2588* was upregulated in both profiles. *Tsac_2588* encodes inosine-5′-monophosphate dehydrogenase (IMPDH), which catalyzes the transformation of inosine monophosphate to XMP with NAD^+^. This reaction not only is the first step in GMP synthesis but also is the rate-limiting process^49^. IMPDH plays a major role in regulation of intercellular GMP, DNA, and RNA synthesis^50^; signal transduction^51^; and membrane glycoprotein synthesis^52^. The regulations in both profiles indicated the role of GMP in DNA repair initiation. *Tsac_2813*, another DNA repair-related gene, was upregulated in the LA1002 versus G40 profile and encoded single-strand binding proteins (SSBs), which can increase the specificity of DNA polymerase against heat treatment^53^ and prevent premature annealing^54^. They considerably affect DNA replication, repair, and recombination in bacteria^55^. As indicated by the comet assay results, DNA repair-related genes are activated, because high osmolality promotes DNA damage to nucleus pulposus cell^56^.

#### Other altered pathways in the two transcriptional profiles

In the SCUT27/*Δldh* versus G58 profile, glycerol consumption was inhibited through the downregulation of *Tsac_1205* and *Tsac_0493* (Fig. 4d). These genes encode the enzymes of glycerol-3-phosphate dehydrogenase (mtGDP), thereby catalyzing glycerophospholipid oxidation for subsequent glycolysis^57, 58^. The downregulation of mtGDP resulted in intracellular glycerol accumulation, which counteracts high substrate conditions^59, 60^.

In the LA1002 versus G40 profile, *Tsac_0296*, *Tsac_1788*, *Tsac_1792*, and *Tsac_0296* encoding flagellar structure proteins were all upregulated more than four times according to the RNA sequencing results (Fig. 4e). Previous reports^61, 62^ emphasized that the genes involved in flagellation are activated by regulator genes, such as those in *Salmonella enterica* serovar Typhi, under hyperosmotic conditions.

## Methods

### Strains and culture conditions

Table 4 shows the bacteria used in this study. The engineered strain *T*. *aotearoense *SCUT27*/Δldh*, in which the gene encoding lactate dehydrogenase was deleted to increase ethanol yield, was obtained and preserved^11^. Normal cell culture was described in the references^11, 12, 23^, and 1 g/L of yeast extract was added into the medium during adaptive domestication to provide abundant amino acid sources. A total of 5 mL of stock culture was activated by transferring it into 125 mL of serum bottles, each of which contained 50 mL of fresh modified MTC medium and incubated at 55 °C and with a nitrogen gas headspace.

**Table 4:** Strains used in this study.

Strains	Description	Source
*T*. *aotearoense* SCUT27/*Δldh*	*Δldh*	Constructed in our previous study^11^
*T*. *aotearoense* SCUT27/*Δldh*-G58	*Δldh*, adapted to high sugar medium	This study
*T*. *aotearoense* LA1002	*Δpta*, *Δack*	Constructed in our previous study^19^
*T*. *aotearoense* LA1002-G40	*Δpta*, *Δack*, adapted to high sugar medium	Constructed in our previous study^23^

### Adaptive evolution of *T*. *aotearoense* SCUT27/*Δldh*

*T*. *aotearoense* SCUT27/*Δldh* was first cultured in medium containing 81 g/L of sugar (glucose:xylose = 2:1, g:g) until the DCW reached approximately 0.7 g/L (approach to stationary phase). The saturated culture was then inoculated into fresh MTC media at the ratio of 1:10. The required time (*t*) for bacterium concentration to reach 0.7 g/L was recorded, and the specific growth rate (g/L/h) was calculated as cell density divided by the needed time. When the specific growth rate remained higher than 0.1 g/L · h at three consecutive times, sugar concentration was gradually enhanced from 102 g/L to 120 g/L. A total of 0.2 mL of culture sample was collected at passage 58 and plated on solid DSMZ 640 medium with 2% agar^19^. Clones were selected randomly after streak plating, and the selected clones were incubated at 55 °C for 3 days. Isolated single colonies were then transferred into separate tubes containing modified MTC medium with 120 g/L of sugars to verify the resistance of the clones against high osmotic pressure. The final isolated clone was designated as SCUT27*/Δldh*-G58.

### Flask-fermentation analysis of SCUT27/*Δldh* and G58

For the investigation of the growth characteristics of SCUT27*/Δldh* and G58 cells, they were cultivated in 125 mL serum bottles containing different sugar concentrations (30, 81, and 120 g/L). The cells were sampled every 6 h, and cell density was measured. All experiments were performed in triplicate.

Propagating stability was analyzed every 10 passages after sequential passing on. The final cell density and ethanol production were monitored after incubation with 120 g/L of sugar for 24 h.

### Batch fermentation in 5 L fermenter

Batch fermentation was carried out in a 5 L of BiostatB fermenter (B. Braun, Germany) containing 2 L of medium with 120 g/L of sugar (80 g/L of glucose and 40 g/L of xylose). The saturated cells of SCUT27/*Δldh* or G58 were inoculated into the fermenter at a ratio of 10% (*v/v*) and then incubated at 55 °C and stirred at 150 rpm. The pH of the fermentation broth was maintained at 6.5 through automatic addition of 5 M NaOH^12^. Residual sugars, ethanol, and organic acids were analyzed at specified intervals via high-performance liquid chromatography (HPLC).

### Transcriptome sequencing and qRT-PCR analysis

Start strain of SCUT27/*Δldh* and LA1002 were cultured with 30 g/L mixed sugars (glucose:xylose = 2:1, g:g), and the evolved SCUT27/*Δldh*-G58 and LA1002-G40 were incubated with 120 g/L of carbon sources. When the cell density of OD_600_ reached 0.8–1.0 (in exponential phase), 1.0 OD cell was harvested via centrifugation (12000 rpm × 2 min) at 4 °C for RNA extraction and 200 OD cells for the analysis of intracellular compounds. Total RNA was extracted using RNAprep pure kit (for cell/bacteria, TIANGEN, Beijing, China) according to the manufacturer’s instructions. RNA sequencing and data analysis were performed by Genewiz (Suzhou, China). Given the imperfection and fragmentation of *T*. *aotearoense* SCUT27 genome, which has been recently updated, the genome annotation of *T*. *saccharolyticum* JW/SL-YS485 (CP003184) in NCBI was selected as reference sequence, because the similarity identities of these species is similar by 99%^14^.

Quantitative real time-PCR (qRT-PCR) was implemented according to standard protocol^14^. Primers were designed under the guidance of IDT website (http://www.idtdna.com/primerquest/Home/Index). Annealing temperature of primers was set in the range of 62–66 °C, and the length of amplicon was set at 100 bp (see Supplementary Table S4). cDNAs were reverse-transcribed using 1 μg of extracted RNA as template. PCR reactions were carried out in 400 nM of each specific primer, SYBR Premix Ex Taq II (TliRNaseH Plus), and 1 μL of diluted cDNA (~100 ng/μL) in a final volume of 20 μL. PCR reactions were run on LightCycler 96 (Roche, Basel, Switzerland) with 30-s incubation at 95 °C, followed by 45 cycles of 95 °C (5 s) and 60 °C (5 s). Sterile Milli-Q water and 16 s rRNA were designed as background control and internal reference, respectively. Three biological replicates were done for each gene, and the results were analyzed via LightCycler 96 SW 1.0 software (Roche).

### Analytical methods

The residual sugars and fermentation products were analyzed via HPLC (Waters 2695, Milford, MA) equipped with an Aminex 87H column (Bio-Rad, Hercules, CA), and a refractive index detector (Waters 2414, Milford, MA). The mobile phase was 5 mM H_2_SO_4_ at a flow rate of 0.6 mL/min. The detector and column temperature were set at 40 and 60 °C, respectively. All the samples were passed through 0.22 μm filters before loading.

UDP-glucose and amino acids in SCUT27*/Δldh* and its derivatives were extracted and measured. Cells were collected and resuspended in 4 mL 20 mM phosphate buffer. After sonication by high-pressure cell disruptor (Constant Systems 01/40/AA, England), the supernatants were collected by centrifugation at 10,000 × g, 4 °C for 30 min and stored at −20 °C.

For amino acid assay, the samples were treated according to the Standard Press of China (NY/T 887–2010), and monitored using amino acid analyzer (L8900, Hitachi, Japan). For UDP-glucose determination, the collected liquid were gently mixed with a triple volume of 95% ethanol and centrifuged at 10,000 × g, 4 °C for 5 min for protein and polysaccharide removal^63^. The supernatants were dried at 55 °C and re-dissolved in water and analyzed by HPLC (Shimadzu CBM-20A, Kyoto, Japan) equipped with a Inertsil NH_2_ column (Shimadzu, Kyoto, Japan), and a diode array detector (Shimadzu SPD-M20A, Kyoto, Japan). The mobile phase was acetonitrile-0.125 M potassium phosphate buffer (60:40, v/v, pH = 3.6) at a flow rate of 1.0 mL/min. The column temperature was set at 30 °C, and the detection wavelength was 262 nm.

Bacterial DCW was determined through a linear correlation equation from the optical density at 600 nm [DCW(g/L) = 0.0371 + 0.3343 × OD_600_]^19^. OD_600_ was monitored using a spectrophotometer (Thermo Fisher Scientific GENESYS 10, Bremen, Germany).

### Data Availability

All data supporting the conclusions of this article are included in this published article and its supplementary information files.

## Electronic supplementary material

Supplementary Table
