# Supplementary material for: Dynamic cell responses in Thermoanaerobacterium sp. under hyperosmotic stress
Source: Sci Rep. 2017 Aug 30;7:10088. doi: 10.1038/s41598-017-10514-8 (PMC5577258; doi:10.1038/s41598-017-10514-8)
Supplement: Supplementary file 1 — Supplementary Table [file 41598_2017_10514_MOESM1_ESM.pdf]

# Dynamic cell responses in *Thermoanaerobacterium* sp. under hyperosmotic stress

Muzi Zhu[1], Wudi Fan[1], Yaping Cha[1], Xiaofeng Yang[1], Zhicheng Lai[1], Shuang Li[1, *], and Xiaoning Wang[2]

**Supplementary Table S1 Comparison of the product accumulation of SCUT27/*Δldh* and G58 at 48 h after inoculation.**

| | Total sugar[a] | | Ethanol | | Acetic acid | |
|---|---|---|---|---|---|---|
| Strains | Original (g/L) | Residual (g/L) | Production (g/L) | Yield (mol/mol sugar) | Production (g/L) | Yield (mol/mol sugar) |
| SCUT27/*Δldh* | 30 | 11.14±1.53 | 4.78±0.26 | 1.10 | 2.23±0.06 | 0.40 |
| | 81 | 65.13±0.10 | 2.33±0.32 | 0.67 | 1.33±0.15 | 0.30 |
| | 120 | 106.42±0.01 | 1.14±0.10 | 0.59 | 0.82±0.01 | 0.33 |
| G58 | 30 | 9.63±0.01 | 4.98±0.02 | 0.98 | 2.54±0.01 | 0.38 |
| | 81 | 64.71±0.10 | 4.84±0.30 | 0.98 | 2.49±0.23 | 0.39 |
| | 120 | 104.52±0.02 | 5.77±0.28 | 0.97 | 2.53±0.10 | 0.33 |

[a] The concentration ratio of glucose and xylose is 2:1 (g:g).

**Supplementary Table S2 The RNA sequencing results of SCUT27/*Δldh* vs G58 (|log(change fold)| ≥ 2.0, FDR ≤ 0.05).**

| Gene locus | Change fold | PValue | FDR [a] |
|---|---|---|---|
| Tsac_2568 | 0.052 | 1.36E-08 | 5.43E-06 |
| Tsac_2565 | 0.076 | 4.24E-07 | 0.000101617 |
| Tsac_1088 | 0.087 | 8.70E-05 | 0.006510443 |
| Tsac_2566 | 0.089 | 2.23E-06 | 0.000380926 |
| Tsac_0897 | 0.098 | 3.46E-05 | 0.003449743 |
| Tsac_2564 | 0.100 | 8.61E-06 | 0.00121222 |
| Tsac_0921 | 0.118 | 2.09E-05 | 0.00238108 |
| Tsac_1631 | 0.130 | 0.000537282 | 0.026250055 |
| Tsac_2145 | 0.137 | 5.04E-05 | 0.004308649 |
| Tsac_1630 | 0.158 | 0.00047154 | 0.024018444 |
| Tsac_0922 | 0.159 | 0.000230571 | 0.013799677 |
| Tsac_2268 | 0.171 | 0.000249494 | 0.014568037 |
| Tsac_0493 | 0.174 | 0.000455703 | 0.023716395 |
| Tsac_1486 | 0.182 | 0.000375905 | 0.020928282 |
| Tsac_2143 | 0.187 | 0.001045831 | 0.044709289 |
| Tsac_1205 | 0.189 | 0.000965149 | 0.04261477 |
| Tsac_0766 | 0.193 | 0.00120826 | 0.049026668 |
| Tsac_2454 | 0.202 | 0.000979036 | 0.04261477 |
| Tsac_1704 | 0.209 | 0.001069741 | 0.044929103 |
| Tsac_2139 | 0.213 | 0.001172807 | 0.048408603 |
| Tsac_2029 | 5.142 | 0.000584735 | 0.027997121 |
| Tsac_0148 | 5.410 | 0.000443438 | 0.023590904 |
| Tsac_0084 | 5.642 | 0.000428385 | 0.023308053 |

| | | | |
|---|---|---|---|
| Tsac_1296 | 5.778 | 0.000265354 | 0.015125171 |
| Tsac_1297 | 6.491 | 0.000110139 | 0.007126277 |
| Tsac_0083 | 6.519 | 0.000103524 | 0.006884349 |
| Tsac_0149 | 7.613 | 6.00E-05 | 0.004954275 |
| Tsac_0150 | 7.974 | 0.000174633 | 0.010719798 |
| Tsac_1295 | 7.991 | 2.27E-05 | 0.00238108 |
| Tsac_2231 | 8.017 | 9.76E-05 | 0.006674674 |
| Tsac_2300 | 8.099 | 4.56E-05 | 0.004043872 |
| Tsac_0151 | 8.140 | 2.00E-05 | 0.00238108 |
| Tsac_2301 | 9.351 | 6.24E-05 | 0.004979371 |
| Tsac_2584 | 11.145 | 1.75E-06 | 0.000321382 |
| Tsac_2232 | 11.381 | 6.52E-06 | 0.000975365 |
| Tsac_2583 | 12.723 | 1.74E-06 | 0.000321382 |
| Tsac_2302 | 13.059 | 4.50E-05 | 0.004043872 |
| Tsac_2238 | 14.382 | 2.18E-05 | 0.00238108 |
| Tsac_2581 | 15.531 | 1.25E-07 | 3.32E-05 |
| Tsac_1294 | 15.546 | 8.52E-08 | 2.55E-05 |
| Tsac_2230 | 18.749 | 9.50E-05 | 0.006674674 |
| Tsac_0939 | 20.695 | 6.15E-09 | 3.68E-06 |
| Tsac_1266 | 20.794 | 0.000621965 | 0.02919579 |
| Tsac_2242 | 21.655 | 0.000488131 | 0.024345531 |
| Tsac_2582 | 21.804 | 1.62E-08 | 5.54E-06 |
| Tsac_2303 | 22.852 | 2.29E-05 | 0.00238108 |
| Tsac_2215 | 23.974 | 1.79E-09 | 1.43E-06 |
| Tsac_2240 | 29.234 | 3.49E-06 | 0.000556826 |
| Tsac_0907 | 30.274 | 6.91E-05 | 0.005338865 |
| Tsac_2304 | 35.311 | 1.70E-10 | 2.03E-07 |

| | | | |
|---|---|---|---|
| Tsac_2588 | 39.984 | 5.09E-11 | 1.22E-07 |
| Tsac_2239 | 57.954 | 1.22E-08 | 5.43E-06 |
| Tsac_1518 | 134.811 | 0.000726851 | 0.033463107 |

[a] False discovery rate.

**Supplementary Table S3 The RNA sequencing results of LA1002 vs G40 (|log(change fold)| ≥ 2.0, FDR ≤ 0.05).**

| Gene locus | Change fold | PValue | FDR |
|---|---|---|---|
| Tsac_1936 | 0.006 | 0.0002906 | 0.0072705 |
| Tsac_2182 | 0.009 | 0.001996 | 0.0280575 |
| Tsac_2500 | 0.035 | 3.01E-10 | 4.73E-08 |
| Tsac_1518 | 0.086 | 1.18E-05 | 0.0005924 |
| Tsac_2508 | 0.108 | 8.31E-05 | 0.0032615 |
| Tsac_1344 | 0.108 | 0.0002915 | 0.0072705 |
| Tsac_1488 | 0.117 | 1.19E-05 | 0.0005924 |
| Tsac_1487 | 0.117 | 1.20E-05 | 0.0005924 |
| Tsac_0241 | 0.135 | 4.88E-05 | 0.0020661 |
| Tsac_0104 | 0.137 | 4.20E-05 | 0.0018371 |
| Tsac_2624 | 0.159 | 0.000131 | 0.0044012 |
| Tsac_1517 | 0.160 | 0.0001534 | 0.0047434 |
| Tsac_0242 | 0.161 | 0.0001485 | 0.0047197 |
| Tsac_1486 | 0.162 | 0.0001544 | 0.0047434 |
| Tsac_2504 | 0.166 | 0.0024703 | 0.0321624 |
| Tsac_2452 | 0.172 | 0.000286 | 0.0072705 |
| Tsac_0041 | 0.184 | 0.0003909 | 0.0086838 |
| Tsac_0691 | 0.184 | 0.0003937 | 0.0086838 |
| Tsac_1323 | 0.187 | 0.0004323 | 0.0093807 |
| Tsac_0692 | 0.189 | 0.0004747 | 0.0099764 |
| Tsac_0227 | 0.198 | 0.0008478 | 0.0149857 |
| Tsac_0042 | 0.204 | 0.0008223 | 0.0148297 |
| Tsac_1515 | 0.209 | 0.0010123 | 0.0169931 |

| | | | |
|---|---|---|---|
| Tsac_2422 | 0.217 | 0.001536 | 0.0232934 |
| Tsac_0812 | 0.233 | 0.0020653 | 0.0284135 |
| Tsac_1389 | 0.237 | 0.002243 | 0.0305041 |
| Tsac_0419 | 0.237 | 0.0022546 | 0.0305041 |
| Tsac_2423 | 0.247 | 0.003742 | 0.0441917 |
| Tsac_1792 | 4.008 | 0.0031621 | 0.0390721 |
| Tsac_1788 | 4.027 | 0.0031385 | 0.0389611 |
| Tsac_1785 | 4.065 | 0.0031303 | 0.0389611 |
| Tsac_1878 | 4.081 | 0.0040842 | 0.047189 |
| Tsac_0565 | 4.189 | 0.0023451 | 0.0309857 |
| Tsac_1793 | 4.302 | 0.0019974 | 0.0280575 |
| Tsac_2557 | 4.320 | 0.0019089 | 0.0276241 |
| Tsac_0593 | 4.395 | 0.0019147 | 0.0276241 |
| Tsac_2296 | 4.424 | 0.0017466 | 0.0256129 |
| Tsac_0789 | 4.449 | 0.0015791 | 0.0236779 |
| Tsac_2813 | 4.466 | 0.0015582 | 0.0234958 |
| Tsac_0628 | 4.560 | 0.0013321 | 0.0210864 |
| Tsac_2283 | 4.593 | 0.0014769 | 0.022525 |
| Tsac_0566 | 4.649 | 0.001168 | 0.0188937 |
| Tsac_0603 | 4.767 | 0.0011067 | 0.0181214 |
| Tsac_1570 | 4.808 | 0.0009789 | 0.016684 |
| Tsac_0564 | 4.816 | 0.0009168 | 0.015786 |
| Tsac_0569 | 4.834 | 0.0008918 | 0.0156212 |
| Tsac_0348 | 4.912 | 0.0008335 | 0.0149306 |
| Tsac_1477 | 4.934 | 0.0007887 | 0.0144189 |
| Tsac_0563 | 5.032 | 0.0006795 | 0.0131426 |
| Tsac_2137 | 5.071 | 0.0006768 | 0.0131426 |

| | | | |
|---|---|---|---|
| Tsac_2540 | 5.091 | 0.0008462 | 0.0149857 |
| Tsac_1563 | 5.156 | 0.0005767 | 0.0116236 |
| Tsac_2588 | 5.176 | 0.0005565 | 0.0114255 |
| Tsac_0568 | 5.192 | 0.0005411 | 0.0111949 |
| Tsac_1375 | 5.209 | 0.0011347 | 0.0184669 |
| Tsac_2189 | 5.300 | 0.0006015 | 0.0118048 |
| Tsac_1475 | 5.530 | 0.0003663 | 0.0084324 |
| Tsac_0597 | 5.592 | 0.0003392 | 0.0080764 |
| Tsac_1745 | 5.637 | 0.0003012 | 0.0074443 |
| Tsac_1671 | 5.666 | 0.0003419 | 0.0080764 |
| Tsac_2297 | 5.741 | 0.0004735 | 0.0099764 |
| Tsac_2584 | 5.792 | 0.0002479 | 0.0067522 |
| Tsac_0599 | 5.801 | 0.0002637 | 0.0071099 |
| Tsac_0296 | 6.270 | 0.0001476 | 0.0047197 |
| Tsac_1678 | 6.389 | 0.0001254 | 0.0043522 |
| Tsac_1476 | 6.937 | 7.35E-05 | 0.0029283 |
| Tsac_2285 | 7.052 | 6.49E-05 | 0.0026264 |
| Tsac_0601 | 7.110 | 9.62E-05 | 0.0036681 |
| Tsac_0294 | 7.137 | 6.33E-05 | 0.0026264 |
| Tsac_0472 | 7.143 | 0.0022661 | 0.0305041 |
| Tsac_2190 | 7.687 | 3.14E-05 | 0.001398 |
| Tsac_1538 | 8.849 | 1.15E-05 | 0.0005924 |
| Tsac_2391 | 11.055 | 1.70E-06 | 0.0001067 |
| Tsac_0477 | 11.176 | 2.28E-06 | 0.0001325 |
| Tsac_0329 | 11.689 | 7.73E-06 | 0.0004124 |
| Tsac_1917 | 12.280 | 6.78E-07 | 5.03E-05 |
| Tsac_2392 | 47.457 | 5.29E-12 | 1.18E-09 |

| | | | |
|---|---|---|---|
| Tsac_1502 | 124.878 | 0.001409 | 0.0217373 |
| Tsac_0092 | 133.137 | 0.0010064 | 0.0169931 |
| Tsac_0085 | 2609.443 | 1.01E-27 | 2.68E-24 |

**Supplementary Table S4 Primers used in this study.**

| Primers | Sequence (from 5' to 3' ends) | Description |
|---|---|---|
| 16s-qU | GCGGAGCATGTGGTTTAATTC | Forward primer for 16s-rRNA in RT-PCR. |
| 16s-qD | CTGTCTCACAGCTCCTCTTTC | Reverse primer for 16s-rRNA in RT-PCR. |
| 1205-U | GGCATTGAATCACCTGGTTTC | Forward primer for Tsac_1205 in RT-PCR. |
| 1205-D | CGGGCTTTCTCTTGGGATTA | Reverse primer for Tsac_1205 in RT-PCR. |
| 0150-U | ACAAGAGGAACCGTCACAAATA | Forward primer for Tsac_0150 in RT-PCR. |
| 0150-D | CCATTACTTGGAGATGGACAGG | Reverse primer for Tsac_0150 in RT-PCR. |
| 1295-U | GGCAAGGAATTGGATGCTTTAG | Forward primer for Tsac_1295 in RT-PCR. |
| 1295-D | TTGACGATGCTTACGGTACAT | Reverse primer for Tsac_1295 in RT-PCR. |
| 1296-U | TGGGAAATGGAGAAGGGTTTAC | Forward primer for Tsac_1296 in RT-PCR. |
| 1296-D | GCTACCAGTTTAGCAGGATCTC | Reverse primer for Tsac_1296 in RT-PCR. |
| 2231-U | ATGCAGATGCTCCTGCTATC | Forward primer for Tsac_2231 in RT-PCR. |

| | | |
|---|---|---|
| 2231-D | CAGGCAAGGGATATGGGTATAAA | Reverse primer for Tsac_2231 in RT-PCR. |
| 2029-U | CTATGAAAGACTGGGCCATTGA | Forward primer for Tsac_2029 in RT-PCR. |
| 2029-D | CCGTCTGGTGTAGGAGAGATAA | Reverse primer for Tsac_2029 in RT-PCR. |
| 0151-U | GCTACAGTTGTTGCCACATATC | Forward primer for Tsac_0151 in RT-PCR. |
| 0151-D | AGAAGATGGTAGATGCAGGAATAG | Reverse primer for Tsac_0151 in RT-PCR. |
| 1294-U | TGGTGATGGTGTGTATGAGATG | Forward primer for Tsac_1294 in RT-PCR. |
| 1294-D | GAGCTCCTCATGTGGATGAAA | Reverse primer for Tsac_1294 in RT-PCR. |
| 2232-U | GCTCCCATACCTGAAGCTATTT | Forward primer for Tsac_2232 in RT-PCR. |
| 2232-D | TGTTCTACTAAGCGGAGTACCT | Reverse primer for Tsac_2232 in RT-PCR. |
| 1476-U | CGCCTTAAAGCGAGATGATTTG | Forward primer for Tsac_1476 in RT-PCR. |
| 1476-D | CACTGTGTATAGCGACCTCTTC | Reverse primer for Tsac_1476 in RT-PCR. |
| 0599-U | CAAAGCATACACCAGAGCATTAC | Forward primer for Tsac_0599 in RT-PCR. |
| 0599-D | CCTGCCTTATGCCGTATACAT | Reverse primer for Tsac_0599 in RT-PCR. |

| | | |
|---|---|---|
| 2297-U | GCCAGTCCAACCTGGTAAAT | Forward primer for Tsac_2297 in RT-PCR. |
| 2297-D | GAAGGAATTGGCATTGGTTCAG | Reverse primer for Tsac_2297 in RT-PCR. |
| 1517-U | TGGCAAACCAGATCCAGAAA | Forward primer for Tsac_1517 in RT-PCR. |
| 1517-D | AGCTTCGATTCCAGCTTTAGAA | Reverse primer for Tsac_1517 in RT-PCR. |
| 2392-U | GGACCTGATGATGAAGGTACTTATT | Forward primer for Tsac_2392 in RT-PCR. |
| 2392-D | GATTGGCTGATGACCATTCTCT | Reverse primer for Tsac_2392 in RT-PCR. |
| 2588-U | TTCTACTTCACCAGGGCTTTC | Forward primer for Tsac_2588 in RT-PCR. |
| 2588-D | GTACAGTGGAGACATCGTCAAA | Reverse primer for Tsac_2588 in RT-PCR. |
| 0104-U | GTATGGGTATGTCCGCCAAATA | Forward primer for Tsac_0104 in RT-PCR. |
| 0104-D | TGGGAGCTACGACTGATAAGA | Reverse primer for Tsac_0104 in RT-PCR. |
| 0564-U | GAAAGCCTTTGAGACAGCATTAG | Forward primer for Tsac_0564 in RT-PCR. |
| 0564-D | GATCCGTTTCTGTCTCCTCTTT | Reverse primer for Tsac_0564 in RT-PCR. |
| 0566-U | CTGACCACTTTGTCCCGAATAA | Forward primer for Tsac_0566 in RT-PCR. |

| | | |
|---|---|---|
| 0566-D | CCATTTGCCCTACCTCAAAGA | Reverse primer for Tsac_0566 in RT-PCR. |
| 0569-U | TGACGGACTTGTGCTTATACC | Forward primer for Tsac_0569 in RT-PCR. |
| 0569-D | CATCGGACCTCCGCTTATTAC | Reverse primer for Tsac_0569 in RT-PCR. |
| 2182-U | TTCTTGTAGGTCATGCCTCTTG | Forward primer for Tsac_2182 in RT-PCR. |
| 2182-D | AACACTGGGTCCTGCATTAG | Reverse primer for Tsac_2182 in RT-PCR. |
| 0628-U | GAAGGCGCTACTGTATGCTATAA | Forward primer for Tsac_0628 in RT-PCR. |
| 0628-D | CTGCCATTACCTCATCGTAGTC | Reverse primer for Tsac_0628 in RT-PCR. |
| 0691-U | AGCTCTGGATATACGGGAGTTA | Forward primer for Tsac_0691 in RT-PCR. |
| 0691-D | TGCTTCCATCTGGCACTAAATA | Reverse primer for Tsac_0691 in RT-PCR. |
| 0227-U | GTAAGCGGGATCCCTACAATAG | Forward primer for Tsac_0227 in RT-PCR. |
| 0227-D | ACAAACCGTCCACAGAAATAAAG | Reverse primer for Tsac_0227 in RT-PCR. |
| 2504-U | CAAACCGACTTTCGTGCTATTT | Forward primer for Tsac_2504 in RT-PCR. |
| 2504-D | TAACGGCATCATTGGAGGAG | Reverse primer for Tsac_2504 in RT-PCR. |

| | | |
|---|---|---|
| 2391-U | CCCATCCAAACACTACCTACATAA | Forward primer for Tsac_2391 in RT-PCR. |
| 2391-D | CCTAATATGCCTGTTGCTGGTA | Reverse primer for Tsac_2391 in RT-PCR. |
| 1570-U | GCTTTCCAGGAGGATGTGATATAG | Forward primer for Tsac_1570 in RT-PCR. |
| 1570-D | CGATTTGCTTTCGCAGTTGAT | Reverse primer for Tsac_1570 in RT-PCR. |
| 1487-U | GTAGTGGGAGGTGGATTGTTAG | Forward primer for Tsac_1487 in RT-PCR. |
| 1487-D | ATCCTCGAACCTTCTTCATCAG | Reverse primer for Tsac_1487 in RT-PCR. |
